# Parathyroid Hormone Levels as an Independent Predictor of Ischemic Heart Disease in Stage 3–5 Non-Dialysis Chronic Kidney Disease: A Retrospective Cohort Study

**DOI:** 10.3390/jcm14103311

**Published:** 2025-05-09

**Authors:** Suthiya Anumas, Pichaya Tantiyavarong, Pattharawin Pattharanitima

**Affiliations:** 1 Chulabhorn International College of Medicine, Thammasat University, Rangsit 12120, Pathum Thani, Thailand; beausuth26@gmail.com; 2 Division of Nephrology, Department of Internal Medicine, Faculty of Medicine, Thammasat University, Rangsit 12120, Pathum Thani, Thailand; pichaya_t@tu.ac.th; 3 Department of Clinical Epidemiology, Faculty of Medicine, Thammasat University, Rangsit 12120, Pathum Thani, Thailand

**Keywords:** parathyroid hormone (PTH), chronic kidney disease (CKD), ischemic heart disease (IHD)

## Abstract

**Background**: Chronic kidney disease–mineral and bone disorder (CKD-MBD) is a key contributor to complications, including ischemic heart disease (IHD), which significantly elevates mortality in patients with chronic kidney disease (CKD). This study aims to identify factors associated with IHD risk in pre-dialysis CKD and establish the minimum parathyroid hormone (PTH) threshold necessary to mitigate this risk. **Methods**: We retrospectively analyzed data from CKD stage 3–5ND patients aged over 18 years, followed from 2018 to 2022. IHD was identified using ICD-10 codes. An adjusted Cox regression model and joint modeling analysis were used to assess the association between risk factors and IHD. **Results:** A total of 1210 CKD patients were included in the analysis, with a median follow-up duration of 513.5 days (IQR 189–979). The incidence of IHD was 7.5%. PTH levels ≥166 ng/L (HR 1.87, 95% CI 1.05–3.35, *p* = 0.03) and age ≥65 years (HR 1.68, 95% CI 1.003–2.81, *p* = 0.04) were significantly associated with an increased risk of IHD. In joint modeling analysis, time-varying PTH, age ≥65 years, and diabetes mellitus (DM) were significantly associated with an increased risk of IHD, whereas ARB and statin use were associated with a reduced risk. Calcium and phosphate levels did not demonstrate significant associations with IHD risk. **Conclusions:** Baseline PTH levels ≥166 ng/L and time-varying PTH were independently and significantly associated with an increased risk of IHD. In contrast, calcium and phosphate levels showed no significant association with IHD risk.

## 1. Introduction

Chronic kidney disease (CKD) affects nearly 10% of the global population [1] and is a major driver of multi-organ complications, particularly within the cardiovascular and neurological systems, thereby amplifying mortality risks [2]. Patients with CKD exhibit an elevated risk of cardiovascular events from stages 1 to 3, with this risk significantly heightened in the advanced stages (CKD stages 4–5) [3,4]. CVD risk in CKD patients is influenced by a complex interplay of traditional and CKD-specific risk factors. Traditional risk factors, well-established in the general population, include advanced age, male gender, hypertension, diabetes mellitus, dyslipidemia, smoking, obesity, and a family history of heart disease [5]. A critical CKD-specific risk factor is chronic kidney disease–mineral and bone disorder (CKD-MBD), with a strong theoretical link to increased vascular calcification in both large and small vessels, heightening the risk of adverse cardiovascular outcomes [6,7]. Some studies report that both parathyroid hormone (PTH) and phosphorus are associated with cardiovascular events [8], while others indicate that only PTH levels are independently linked to these events [9,10]. Consequently, the consensus remains controversial. Nevertheless, early detection and increased awareness of CKD-MBD biomarkers are crucial for reducing morbidity and mortality associated with this condition.

The management of CKD-MBD, particularly in patients with non-dialysis CKD, remains a significant challenge for clinicians due to the complex interactions among various factors and the lack of well-defined guidelines for certain clinical parameters. The Kidney Disease: Improving Global Outcomes (KDIGO) guidelines recommend monitoring serum levels of calcium, phosphate, PTH, and alkaline phosphatase activity beginning in CKD stage G3a [11]. This recommendation has been consistent across different versions of the guidelines, including the 2009 and 2017 updates [12]. While all the guidelines, including those from the recent controversial KDIGO conference, provide recommendations for monitoring, they do not specify cut-off PTH levels for non-dialysis CKD patients, in contrast to those for dialysis patients [11,13]. However, the Kidney Disease Outcomes Quality Initiative (KDOQI) 2003 [14] guideline establishes target ranges for PTH levels in CKD stage 3–5D. For stages 3 and 4, the recommendations are primarily based on expert opinion rather than empirical evidence, reflecting the limited data available for non-dialysis CKD patients at that time.

This study aims to identify risk factors associated with CKD, with a particular focus on ischemic heart disease (IHD). Specifically, the study seeks to determine the approximate PTH cut-off level that correlates with an increased risk of IHD, in order to provide guidance for clinical practice. Additionally, considering recent advancements in pharmacotherapy with evidence supporting a reduction in CVD risk, our analysis will also incorporate the impact of these therapeutics within the model.

## 2. Materials and Methods

### 2.1. Study Population and Clinical Data

This study included patients with CKD stage 3–5ND without established IHD over a five-year period from 1 January 2018 to 31 December 2022. A total of 1210 patients were analyzed. CKD was defined and classified according to the KDIGO 2024 guidelines [15]. New diagnoses of IHD during the follow-up period were identified based on ICD-10 codes (Table S1).

Baseline demographic data, including age, sex, blood pressure, and body weight were obtained from electronic medical records. Laboratory results, creatinine, hemoglobin, corrected calcium, phosphorus, intact parathyroid hormone (intact-PTH), serum albumin, and lipid profile, were recorded as baseline values. The estimated glomerular filtration rate (eGFR) was calculated using the CKD-EPI 2021 formula. Medication use was documented if patients had a history of using the medication for at least six months. All patients were monitored for the occurrence of IHD outcomes during their participation in the cohort.

We utilized tertiles of the data to determine the cut-off levels for PTH. Additionally, we performed an analysis using the cut-off levels for PTH recommended by the KDOQI 2003 guidelines [14]. According to these guidelines, the target PTH levels are 35–70 ng/L for CKD stage 3, 70–110 ng/L for CKD stage 4, and 150–300 ng/L for CKD stage 5 or dialysis. PTH levels exceeding these target ranges were classified as “high PTH”. For corrected calcium levels, values exceeding 10 mg/dL were classified as hypercalcemia. Phosphate levels greater than 4.5 mg/dL were considered indicative of hyperphosphatemia.

### 2.2. Study Endpoints

The primary endpoints were the identification of risk factors and the determination of the PTH cut-off associated with IHD in patients with CKD stage 3–5 non-dialysis (CKD 3–5ND). Additionally, we analyzed the associated parameters across four models, incorporating key adjustment factors. As a secondary outcome, the PTH cut-off recommended by KDOQI was reported in our model and compared to our own PTH tertile cut-off for patients with CKD stage 3–5ND. Furthermore, we analyzed the effect of changes in PTH levels over time (time-varying PTH) and their association with IHD.

### 2.3. Statistical Analysis

Continuous variables were reported as mean  ±  standard deviation (SD) or as median with interquartile range (IQR), depending on the data’s distribution, and were compared using either an unpaired t-test or a Mann–Whitney U test, as applicable. Categorical variables were presented as frequencies and percentages and were analyzed using a Chi-square test or Fisher’s exact test for comparisons. The relationship between CKD-MBD biomarkers and the risk of IHD was evaluated using Cox proportional hazards models, with adjustments made for potential confounders in a sequential manner. The validity of the proportional hazards assumption was tested using the Schoenfeld test. Effect modification was investigated by applying various models: Model 1 included intact-PTH, corrected calcium, and phosphorus; Model 2 incorporated non-modifiable risk factors such as age and sex into Model 1; Model 3 added diabetes and hypertension to Model 2; and the final model included medication use in addition to the variables in Model 3.

A joint modeling approach was also applied, integrating a longitudinal submodel and a survival submodel, with coefficients presented for interpretation. The longitudinal submodel employed a linear mixed-effects model to investigate the association of time-varying PTH levels over time. The survival submodel used a Weibull proportional hazards model to evaluate the time-to-event outcome. Two joint submodels were constructed: a univariable model including time-varying PTH and a multivariable model incorporating the same covariates as the final Cox proportional hazards model. Statistical analysis was performed using Stata 17.0/BE (College Station, TX, USA).

## 3. Results

### 3.1. Patient Characteristics

**Figure 1:**
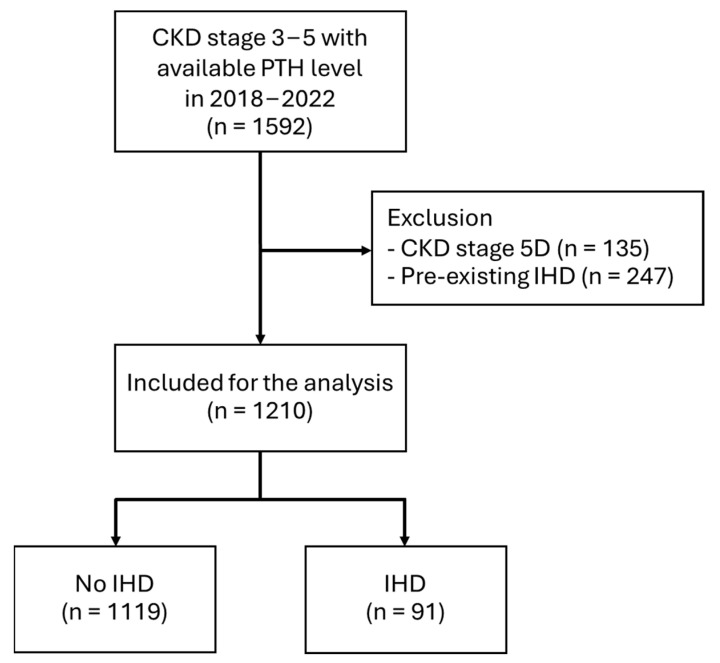
Flow of the study. Abbreviations: CKD, chronic kidney disease; D, dialysis, IHD, ischemic heart disease; PTH, parathyroid hormone.

Among the 1592 CKD stage 3–5 patients with available PTH levels screened, 1210 patients met the inclusion criteria and were included in the analysis. The remaining 382 patients were excluded due to pre-existing ischemic heart disease and CKD stage 5 on dialysis (Figure 1). The incidence of IHD in the study population was 7.5%. Median follow-up time was 513.5 (189–979) days. Within the cohort, CKD stage 3a was diagnosed for 23.1%, stage 3b for 25.9%, stage 4 for 22.2%, and stage 5 ND for 28.8%. The incidence of IHD increased progressively with advancing CKD stages (Table 1), with a median time to IHD of 365 days (IQR 126–684).

Patients in the IHD group had a higher prevalence of congestive heart failure (CHF) and exhibited lower diastolic blood pressure (DBP) and body mass index (BMI) compared to those without IHD. Regarding laboratory findings, hemoglobin, serum albumin, and HDL were lower in the IHD group, while PTH levels were elevated. However, no significant differences were observed between the IHD and non-IHD groups in terms of corrected calcium and phosphorus levels. Additionally, patients without IHD demonstrated significantly higher usage of angiotensin receptor blockers (ARBs), sodium–glucose cotransporter-2 (SGLT2) inhibitors, and statins compared to those with IHD (Table 1).

### 3.2. Risk of IHD and Minimum PTH Cut-Off Level

**Figure 2:**
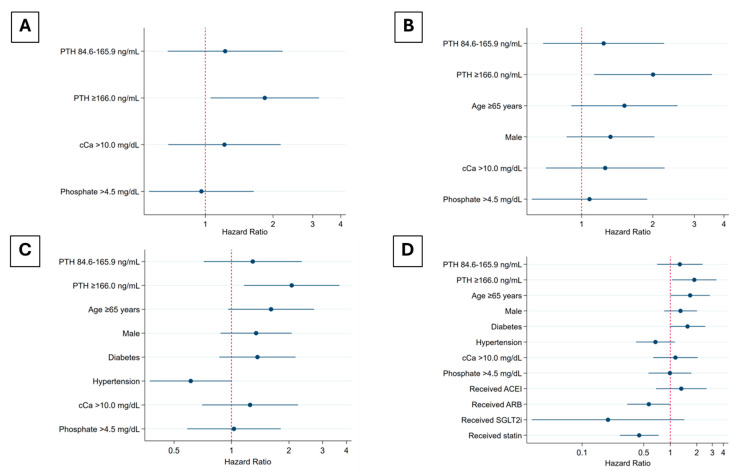
Adjusted HRs for IHD in CKD stage 3–5ND: (**A**) Model 1 (adjusted for corrected calcium >10.0 mg/dL and phosphate >4.5 mg/dL); (**B**) Model 2 (adjusted as Model 1, and age ≥65 years and gender); (**C**) Model 3 (adjusted as Model 2, and diabetes and hypertension); and (**D**) Model 4 (adjusted as Model 3, and received ACEI, ARB, SGLT2i, and statin). Abbreviations: ACEI, angiotensin-converting enzyme inhibitor; ARB, angiotensin receptor blocker; cCa, corrected calcium; CKD, chronic kidney disease; D, dialysis; HR, hazard ratio; IHD, ischemic heart disease; ND, non-dialysis; PTH, parathyroid hormone; SGLT2i, sodium–glucose cotransporter-2 inhibitor.

In the unadjusted analysis, baseline PTH levels ≥166 ng/L were significantly associated with an increased risk of IHD (HR 1.87, 95% CI 1.11–3.13, *p* = 0.02), whereas calcium and phosphate levels demonstrated no significant associations. In the adjusted analyses, Model 1, which accounted for all recorded CKD-MBD parameters, established that baseline PTH levels ≥166 ng/L remained the sole factor significantly associated with IHD (HR 1.84, 95% CI 1.05–3.20, *p* = 0.03). Model 2, incorporating traditional IHD risk factors such as age ≥65 years and sex, reinforced the significant association between baseline PTH levels ≥166 ng/L and IHD (HR 2.01, 95% CI 1.13–3.56, *p* = 0.02). Model 3, which adjusted for additional comorbidities including hypertension and diabetes, confirmed the significance of baseline PTH levels ≥166 ng/L (HR 2.07, 95% CI 1.16–3.68, *p* = 0.01) as a predictive factor. In the final model, with further adjustments for the use of angiotensin-converting enzyme inhibitors (ACEIs), ARBs, sodium–glucose cotransporter-2 inhibitors (SGLT2is), and statins, baseline PTH levels ≥166 ng/L and age ≥65 years were significantly associated with an elevated risk of IHD, while statin use emerged as a protective factor against IHD in patients with CKD stage 3–5ND (Table 2, Figure 2).

### 3.3. Risk of IHD in CKD Stage 3–5ND Using PTH KDOQI Cut-Offs and Tertile Cut-Offs

We also assessed elevated PTH levels according to the KDOQI recommendations for each CKD stage. Our analysis demonstrated that a notable proportion of patients with CKD stage 3–5ND exhibited high PTH levels: 59.3% when applying the KDOQI cut-off, and 40.2% when using the tertile-based PTH cut-off (PTH levels ≥166 ng/L).

**Figure 3:**
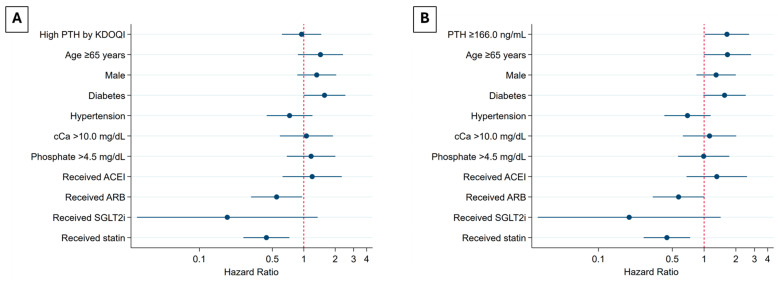
Adjusted HRs for IHD in CKD stage 3–5ND. (**A**) High PTH cut-off levels according to the KDOQI guidelines; and (**B**) PTH cut-off levels according to highest PTH tertile. Abbreviations: ACEI, angiotensin-converting enzyme inhibitor; ARB, angiotensin receptor blocker; cCa, corrected calcium; CKD, chronic kidney disease; D, dialysis; HR, hazard ratio; IHD, ischemic heart disease; KDOQI, Kidney Disease Outcomes Quality Initiative; ND, non-dialysis; PTH, parathyroid hormone; SGLT2i, sodium–glucose cotransporter-2 inhibitor.

In the unadjusted analysis, high PTH levels, as defined by the KDOQI 2003 guidelines, were not significantly associated with IHD, a finding that remained consistent across all adjusted models. However, in the final model, the use of ARBs and statins was significantly associated with a reduced risk of IHD, with HRs of 0.55 (95% CI 0.31–0.96, *p* = 0.04) and 0.44 (95% CI 0.26–0.73, *p* = 0.001), respectively (Table S2, Figure 3A).

When baseline PTH levels were categorized using a tertile-based cut-off (PTH levels ≥166 ng/L), all adjusted models demonstrated that baseline PTH ≥166 ng/L was significantly associated with an increased risk of IHD. In the final model, baseline PTH ≥166 ng/L was identified as a factor increasing the risk of IHD, with a hazard ratio (HR) of 1.65 (95% CI 1.02–2.66, *p* = 0.04). In contrast, statin use was found to be a protective factor, with an HR of 0.44 (95% CI 0.27–0.74, *p* = 0.002) (Table S3, Figure 3B).

### 3.4. Joint Model to Evaluate Time-Varying PTH and the Risk of IHD

We further examined the time-varying effects of PTH on the risk of IHD using a multivariable joint model analysis, incorporating longitudinal PTH data and a survival submodel. The longitudinal submodel demonstrated that PTH levels significantly changed over time (time-varying PTH), with a coefficient of 0.16 (95% CI 0.12–0.19, *p* < 0.001). In the survival submodel, which accounted for time-varying PTH, along with calcium, phosphorus, age ≥65 years, sex, diabetes, hypertension, and medication use, time-varying PTH was significantly associated with an increased risk of IHD (coefficient = 0.001, 95% CI 0.0001–0.002, *p* = 0.022). Additionally, age ≥65 years (coefficient = 0.54, 95% CI 0.02–1.07, *p* = 0.04) and diabetes (coefficient = 0.51, 95% CI: 0.05–0.97, *p* = 0.03) were identified as independent risk factors for IHD. Conversely, the use of ARBs (coefficient = −0.57, 95% CI −1.13 to −0.01, *p* = 0.046) and the use of statins (coefficient = −0.82, 95% CI −1.32 to −0.31, *p* = 0.002) were associated with a significantly reduced risk of IHD (Table 3).

## 4. Discussion

This study demonstrated that PTH levels ≥166 ng/L and age ≥65 years were associated with an increased risk of IHD, while statin use is associated with a decreased risk of IHD in individuals with CKD stage 3–5ND. Consequently, among the identified risk factors, PTH levels should be prioritized as a biomarker for prompting management strategies. Additionally, statins should be considered for all CKD patients to mitigate IHD risk.

The prevalence of IHD observed in our study was 7.5%, compared to 9.8% reported in a large cohort study from China [16], which also included an Asian population. However, the Chinese study predominantly reported cerebrovascular disease as the major cardiovascular condition. Specifically, for myocardial infarction, the reported prevalence was only 2%, which is lower than our findings. Importantly, the Chinese study did not include individuals with CKD stage 5 ND, a group known to have the highest prevalence of IHD among pre-dialysis patients. Compared to the ROUTE study from Japan [17], which included an Asian population with CKD stages 2–5 and reported a 26.8% prevalence of major cardiovascular conditions, including IHD, peripheral arterial disease, stroke, and congestive heart failure, our study focused specifically on IHD. Consequently, the observed prevalence of IHD in our study was much lower than the prevalence of overall major cardiovascular conditions reported in the ROUTE study (7.5% vs. 26.8%). However, our results demonstrate a sequential increase in the prevalence of IHD with advancing stages of CKD, aligning with global reports [3,7,18].

A large Korean cohort study demonstrated that a DBP of 60–69 mmHg is significantly associated with increased IHD mortality, possibly due to impaired coronary perfusion, as myocardial blood flow primarily occurs during diastole [19]. Similarly, our study found that the IHD group had a lower mean DBP compared to the non-IHD group (68.4 vs. 71.9 mmHg). However, low DBP is not universally harmful and should be interpreted alongside SBP. While it may reflect increased cardiovascular risk, current evidence indicates that BP reduction remains beneficial when targeting elevated SBP [20]. Additionally, our study reported lower hemoglobin and serum albumin levels in the IHD group, which aligns with other reports associating these factors with CVD in CKD [21,22]. In our analysis of lipid profiles, we observed that the IHD group exhibited lower high-density lipoprotein (HDL) cholesterol levels compared to the non-IHD group. This finding aligns with the well-established phenomenon of reduced HDL cholesterol levels in patients with CKD. It is noteworthy that, in the context of CKD, HDL particles may undergo functional alterations, potentially losing their crucial anti-atherosclerotic properties and becoming dysfunctional [23,24,25]. These changes may contribute to an increased risk of IHD. However, it is important to note that we did not incorporate DBP, hemoglobin, serum albumin, or HDL levels into our adjusted model. This decision was predicated on the inherent variability of these parameters, which renders single time-point measurements potentially unreliable for robust statistical analysis. Consequently, our findings regarding these factors should be interpreted as observational trends rather than definitive associations.

In our univariate analysis, we identified that PTH levels ≥166 ng/L were associated with an increased risk of IHD. However, we did not observe a significant correlation between hypercalcemia or hyperphosphatemia and the risk of IHD. This lack of correlation contrasts with findings from the NEFRONA cohort [8], which reported that both secondary hyperparathyroidism and hyperphosphatemia were associated with an elevated risk of cardiovascular events. Despite the average phosphorus levels in our study being comparable to those in the NEFRONA study (3.9 mg/dL vs. 3.8 mg/dL), and using the same definition for hyperphosphatemia, our results diverged. To address potential confounding, we conducted a four-model analysis adjusting for non-traditional risk factors, and the results consistently demonstrated that elevated PTH levels ≥166 ng/L and age ≥65 years were associated with an increased risk of IHD. However, our results are consistent with those of Lismanov et al. [9] and Bhuriya et al. [26], who conducted a study in CKD stage 3–4 and reported that only PTH levels were independently associated with cardiovascular disease, irrespective of calcium and phosphorus levels.

We also conducted analysis using a PTH cut-off of ≥166 ng/L across four models and comparing it with PTH cut-offs according to KDOQI guidelines, as used in the NEFRONA study. We found that the results for the PTH cut-off of ≥166 ng/L were consistent with those observed for CKD stage 3–5ND. However, when we applied the KDOQI-based PTH cut-offs, our analysis yielded different results. Specifically, we observed no significant increase in risk associated with any models using KDOQI-based PTH cut-offs. These findings contrast with the NEFRONA study, which reported significant associations between both hyperparathyroidism, by KDOQI-based PTH cut-offs, and hyperphosphatemia and increased cardiovascular disease risk. This discrepancy may be attributed to the difference in baseline PTH levels in our population compared to the NEFRONA study [median PTH 115.5 (70.1–213.1) ng/L vs. 127.8 (71–227.7) ng/L].

The EQUAL study [27], which assessed mortality in CKD stages 4–5 over a five-year follow-up period, found no significant association between baseline PTH levels and cardiovascular mortality. However, time-dependent PTH levels were significantly associated with outcomes. These findings emphasize the importance of considering time-dependent PTH levels when evaluating cardiovascular risk in CKD populations. While our primary outcome analysis focused on baseline CKD-MBD biomarkers, including PTH, calcium, and phosphorus, to determine optimal cut-off levels and their associations with IHD, we acknowledge the need for further investigation into the relationship between time-dependent PTH levels and IHD risk. In our study, we conducted a joint model analysis to assess the time-varying effects of PTH. These findings support the recommendation that a progressive increase in PTH levels should be evaluated [13,26]. Additionally, age ≥65 years and the presence of diabetes were associated with an increased risk of IHD. In contrast, statin use, along with ARB use, was associated with a decreased risk of IHD. These findings underscore the importance of considering both baseline and time-dependent PTH levels in the evaluation of cardiovascular risk in CKD populations.

In the final model of Cox proportional hazards analysis, we incorporated ACEIs, ARBs, SGLT2is, and statins, given their well-established efficacy in reducing cardiovascular events in CKD patients [25,28,29]. After adjusting for these evidence-based therapies, our analysis revealed statistically significant reductions in the risk associated with IHD only for statins. Theoretically, ACEI and ARBs should have efficacy in decreasing cardiovascular events [30,31]. However, our study had a low proportion of ACEI and ARB use, which may have limited our ability to detect a statistically significant association with IHD risk. Similarly, although SGLT2 inhibitors (SGLT2is) have demonstrated cardiovascular benefits in patients with CKD through various mechanisms—such as improving endothelial function and vasodilation, optimizing myocardial energy metabolism, preserving cardiac contractility, and exerting anti-inflammatory effects [28,32,33]—the low usage rate of SGLT2is in our cohort limited our ability to identify a statistically significant association. Nevertheless, the observed trend suggested a potential protective effect.

The use of statins for primary prevention of cardiovascular events is well-established in general populations, including patients with CKD [34]. However, in the context of CKD non-dialysis, the SHARP study [35] is the landmark trial demonstrating that a combination of simvastatin and ezetimibe reduces the risk of major atherosclerotic events. It is noteworthy that the SHARP study did not include a statin monotherapy arm, which limits direct comparisons with statin-only interventions. Our findings align with current recommendations. The observed association between statin use and reduced risk of IHD in our study corroborates the potential cardioprotective effects of statins in CKD patients.

The strengths of our study include its large cohort spanning CKD stage 3–5ND and the use of complete datasets, ensuring accuracy and eliminating reliance on imputative data. We employed a four-model approach with clinically relevant parameters to adjust outcomes, thereby accurately reflecting real-world clinical conditions. Most importantly, our study provides valuable insights into approximate PTH cut-off levels, a critical issue in clinical practice. Furthermore, we utilized joint model analysis, an advanced statistical method particularly suited for evaluating time-to-event data in relation to dynamic variables such as PTH levels and their associated outcomes.

However, several limitations should be acknowledged. Firstly, we lacked vitamin D data due to limited availability of testing in our cohort. Secondly, the diagnosis of ischemic heart disease based solely on ICD-10 codes may introduce diagnostic bias. Further research utilizing definitive diagnostic results, such as those from coronary angiography (CAG), is warranted to validate these findings. Thirdly, the small proportion of our population receiving ACEIs, ARBs and SGLT2is warrants cautious interpretation of these findings. Lastly, as the study was conducted at a single center, the generalizability of the results should be considered.

## 5. Conclusions

Our study identified that a PTH level ≥166 ng/L and age ≥65 years were significant risk factors for IHD in patients with CKD stage 3–5ND, while the use of statins was associated with a reduction in risk. Furthermore, time-varying PTH levels were also significantly associated with an increased risk of IHD. In contrast, calcium and phosphate levels demonstrated no significant association with IHD risk. Therefore, monitoring PTH levels in CKD stage 3–5ND should be considered as a strategy to minimize the risk of IHD.

## Figures and Tables

**Table 1:** Baseline characteristics of CKD patients with and without ischemic heart disease (IHD).

Characteristics	Total	IHD	Non-IHD	*p*-Value
	n = 1210	n = 91	n = 1119	
Age, years, mean (SD)	70.2 (14.4)	70.9 (12.6)	70.2 (14.6)	0.85
Male, n (%)	534 (44.1)	44 (48.3)	490 (43.8)	0.23
BMI, kg/m^2^, mean (SD)	23.9 (4.8)	22.9 (4.7)	24.0 (4.8)	0.03
SBP, mmHg, mean (SD)	136.8 (23.5)	138.3 (24.7)	136.6 (23.2)	0.61
DBP, mmHg, mean (SD)	71.6 (15.4)	68.4 (16.5)	71.9 (15.3)	0.01
Comorbidities, n (%)				
Congestive heart failure	89 (7.4)	13 (14.3)	76 (6.8)	0.01
Hypertension	864 (71.4)	59 (64.8)	805 (71.9)	0.15
Paralysis	52 (4.3)	3 (3.3)	49 (4.4)	0.44
Peripheral vascular disorder	23 (1.9)	2 (2.2)	21 (1.9)	0.53
Diabetes mellitus	555 (45.9)	44 (48.3)	511 (45.7)	0.35
Dyslipidemia	661 (54.6)	44 (48.3)	617 (55.1)	0.23
eGFR, mL/min/1.73 m^2^, mean (SD)	29.4 (17.4)	22.7 (16.8)	29.9 (17.3)	0.001
CKD stage, n (%)				
CKD stage 3a	279 (23.1)	13 (14.3)	266 (23.8)	<0.001
CKD stage 3b	313 (25.9)	13 (14.3)	300 (26.8)	
CKD stage 4	269 (22.2)	24 (26.4)	245 (21.9)	
CKD stage 5	349 (28.8)	41 (45.0)	308 (27.5)	
Hb, g/dL, mean (SD)	10.8 (2.1)	10.2 (1.9)	10.8 (2.1)	0.004
Albumin, g/dL, mean (SD)	3.6 (0.5)	3.4 (0.5)	3.6 (0.5)	<0.001
LDL, mg/dL, mean (SD)	100.0 (37.1)	97.3 (37.4)	100.2 (37.1)	0.36
Triglycerides, mg/dL, mean (SD)	136.7 (81.7)	144.4 (70.2)	136.1 (82.6)	0.14
Cholesterol, mg/dL, mean (SD)	175.8 (52.2)	171.7 (50.1)	176.2 (52.4)	0.35
HDL, mg/dL, mean (SD)	51.6 (16.5)	46.3 (13.0)	52.0 (16.7)	0.008
Calcium (corrected), mg/dL, mean (SD)	9.5 (0.8)	9.4 (0.7)	9.5 (0.8)	0.91
≤10 mg/dL, n (%)	987 (85.4)	73 (83.9)	914 (85.6)	0.38
>10 mg/dL, n (%)	168 (14.5)	14 (16.1)	154 (14.4)	
Phosphate, mg/dL, mean (SD)	3.9 (1.2)	4.0 (1.6)	3.9 (1.2)	0.44
≤4.5 mg/dL, n (%)	954 (81.3)	67 (75.3)	887 (81.8)	0.08
>4.5 mg/dL, n (%)	219 (18.7)	22 (24.7)	197 (18.2)	
PTH, ng/L, median (IQR)	115.5 (70.1–213.1)	144.2 (84.6–287.5)	114.1 (70–209.2)	0.02
<84.6 ng/L, n (%)	403 (33.3)	22 (24.2)	381 (34.1)	0.038
84.6–165.9 ng/L, n (%)	403 (33.3)	28 (30.8)	375 (33.5)	
≥166 ng/L, n (%)	404 (33.4)	41 (45.0)	363 (32.4)	
Medication, n (%)				
SGLT2i	75 (6.2)	1 (1.1)	74 (6.6)	0.02
ACEI	126 (10.4)	11 (12.1)	115 (10.3)	0.34
ARBs	373 (30.8)	16 (17.6)	357 (31.9)	0.002
Statin	459 (37.9)	21 (23.1)	438 (39.1)	0.001

Abbreviations: ACEI, angiotensin-converting enzyme inhibitors; ARBs, angiotensin receptor blockers; BMI, body mass index; CKD, chronic kidney disease; DBP, diastolic blood pressure; eGFR, estimated glomerular filtration rate; Hb, hemoglobin; PTH, parathyroid hormone; SBP, systolic blood pressure; SGLT2i, sodium–glucose cotransporter-2 inhibitor.

**Table 2:** Determinants associated with ischemic heart disease (IHD).

Raw Effect	HR	95% CI	*p*-Value
PTH level, ng/L, n (%)			
<84.6	ref		
84.6–165.9	1.36	0.78–2.38	0.28
≥166	1.87	1.11–3.13	0.02
Hypercalcemia	1.19	0.67–2.11	0.55
Hyperphosphatemia	1.27	0.79–2.06	0.32
**Model 1**	**HR**	**95% CI**	***p*-value**
PTH level, ng/L, n (%)			
<84.6	ref		
84.6–165.9	1.22	0.68–2.20	0.50
≥166	1.84	1.05–3.20	0.03
Hypercalcemia	1.22	0.68–2.16	0.50
Hyperphosphatemia	0.96	0.56–1.64	0.88
**Model 2**	**HR**	**95% CI**	***p*-value**
PTH level, ng/L, n (%)			
<84.6	ref		
84.6–165.9	1.24	0.69–2.23	0.5
≥166	2.01	1.13–3.56	0.02
Hypercalcemia	1.26	0.71–2.24	0.43
Hyperphosphatemia	1.08	0.61–1.89	0.79
Age ≥65 years	1.52	0.90–2.54	0.11
Male	1.32	0.86–2.03	0.20
**Model 3**	**HR**	**95% CI**	***p*-value**
PTH level, ng/L, n (%)			
<84.6	ref		
84.6–165.9	1.29	0.72–2.33	0.39
≥166	2.07	1.16–3.68	0.01
Hypercalcemia	1.25	0.70–2.23	0.45
Hyperphosphatemia	1.03	0.58–1.81	0.92
Age ≥65 years	1.61	0.95–2.71	0.07
Male	1.34	0.88–2.07	0.17
HT	0.61	0.37–1.00	0.051
DM	1.37	0.86–2.17	0.18
**Model 4**	**HR**	**95% CI**	***p*-value**
PTH level, ng/L, n (%)			
<84.6	ref		
84.6–165.9	1.28	0.71–2.32	0.41
≥166	1.87	1.05–3.35	0.03
Hypercalcemia	1.14	0.64–2.05	0.65
Hyperphosphatemia	0.99	0.57–1.73	0.97
Age ≥65 years	1.68	1.00–2.81	0.04
Male	1.31	0.85–2.01	0.22
HT	0.68	0.41–1.13	0.13
DM	1.57	0.99–2.49	0.056
Medication			
ACEI	1.33	0.69–2.58	0.39
ARB	0.57	0.32–1.00	0.051
SGLT2i	0.2	0.03–1.44	0.11
Statin	0.44	0.27–0.73	0.002

Abbreviations: ACEI, angiotensin-converting enzyme inhibitors; ARBs, angiotensin receptor blockers; DM, diabetes mellitus; HT, hypertension; PTH, parathyroid hormone; SGLT2i, sodium–glucose cotransporter-2 inhibitor.

**Table 3:** Coefficients from multivariable joint model analysis demonstrating the association between time-varying PTH and IHD.

Joint Model	Coefficients (95% CI)	*p* Value
Longitudinal submodel		
Intercept	232.75 (217.17 to 248.32)	<0.001
Time	0.16 (0.12 to 0.19)	<0.001
Survival submodel		
Time-varying PTH	0.001 (0.0001 to 0.002)	0.022
Hypercalcemia	−0.04 (0.63 to 0.55)	0.91
Hyperphosphatemia	0.04 (−0.52 to 0.59)	0.9
Age ≥65 years	0.54 (0.02 to 1.07)	0.04
Male	−0.33 (−0.76 to 0.10)	0.14
HT	−0.39 (−0.90 to 0.12)	0.13
DM	0.51 (0.05 to 0.97)	0.03
Medication		
ACEI	0.24 (−0.41 to 0.90)	0.47
ARB	−0.57 (−1.13 to −0.01)	0.046
SGLT2i	−1.71 (−3.70 to 0.29)	0.09
Statin	−0.82 (−1.3 to −0.31)	0.002

Abbreviations: ACEI, angiotensin-converting enzyme inhibitor; ARBs, angiotensin receptor blockers; DM, diabetes mellitus; HT, hypertension; PTH, parathyroid hormone; SGLT2i, sodium–glucose cotransporter-2 inhibitor.

## Data Availability

The data presented in this study are available on request from the corresponding author. The data are not publicly available due to privacy and ethical restrictions.

## Supplementary Materials

The following supporting information can be downloaded at: https://www.mdpi.com/article/10.3390/jcm14103311/s1, Table S1. ICD-10 Classification Used for Identifying Outcomes and Comorbidities. Table S2. Determinants Associated with Ischemic Heart Disease (IHD) in CKD Stage 3–5ND According to PTH KDOQI Cut-offs. Table S3. Determinants Associated with Ischemic Heart Disease (IHD) in CKD Stage 3–5ND According to our Cut-offs (PTH level ≥166 ng/L).
