# Supplementary material for: Parathyroid Hormone Levels as an Independent Predictor of Ischemic Heart Disease in Stage 3–5 Non-Dialysis Chronic Kidney Disease: A Retrospective Cohort Study
Source: J Clin Med. 2025 May 9;14(10):3311. doi: 10.3390/jcm14103311 (PMC12112655; doi:10.3390/jcm14103311)
Supplement: Supplementary file 1 [file jcm-14-03311-s001.zip › jcm-3592504-supplementary.pdf]

Supplementary Tables

Table S1. ICD-10 Classification Used for Identifying Outcomes and Comorbidities

**Diagnosis**	**ICD-10 code**
IHD	I20.x-I25.x
CHF	I09.9, I11.0, I13.0, I13.2, I25.5, I42.0, I42.5 - I42.9, I43.x, I50.x, P29.0
Peripheral vascular disorders	I70.x, I71.x, I73.1, I73.8, I73.9, I77.1, I79.0, I79.2, K55.1, K55.8, K55.9, Z95.8, Z95.9
Hypertension	I10.x, I11.x - I13.x, I15.x
Diabetes	E10.0, E10.1, E10.9, E11.0, E11.1, E11.9, E12.0, E12.1, E12.9, E13.0, E13.1, E13.9, E14.0, E14.1, E14.9, E10.2 - E10.8, E11.2 - E11.8, E12.2 - E12.8, E13.2 - E13.8, E14.2 - E14.8
Paralysis	G04.1, G11.4, G80.1, G80.2, G81.x, G82.x, G83.0 - G83.4, G83.9
Dyslipidemia	E78.x

Reference: Quan H, Sundararajan V, Halfon P, et al. Coding algorithms for defining Comorbidities in ICD-9-CM and ICD-10 administrative data. Med Care. 2005 Nov; 43(11): 1130-9.

Table S2. Determinants Associated with Ischemic Heart Disease (IHD) in CKD Stages 3-5ND According to PTH KDOQI Cut-offs.

**Raw effect**	**HR**	**95% CI**	**p-value**
High PTH	0.85	0.56-1.28	0.44
**Model 1**	**HR**	**95% CI**	**p-value**
High PTH	0.88	0.57-1.35	0.57
Hypercalcemia	1.14	0.64-2.03	0.65
Hyperphosphatemia	1.22	0.74-2.00	0.42
**Model 2**	**HR**	**95% CI**	**p-value**
High PTH	0.89	0.58-1.37	0.60
Hypercalcemia	1.17	0.65-2.08	0.60
Hyperphosphatemia	1.31	0.76-2.25	0.32
Age ≥65 years	1.27	0.77-2.08	0.35
Male	1.38	0.90-2.11	0.14
**Model 3**	**HR**	**95% CI**	**p-value**
High PTH	0.91	0.59-1.41	0.68
Hypercalcemia	1.14	0.64-2.04	0.65
Hyperphosphatemia	1.26	0.73-2.16	0.40
Age ≥65 years	1.33	0.81-2.18	0.26
Male	1.41	0.92-2.16	0.11
HT	0.63	0.39-1.04	0.07
DM	1.36	0.86-2.15	0.19
**Model 4**	**HR**	**95% CI**	**p-value**
High PTH	0.95	0.62-1.47	0.82
Hypercalcemia	1.06	0.59-1.90	0.85
Hyperphosphatemia	1.17	0.69-2.01	0.56
Age ≥65 years	1.44	0.88-2.37	0.15
Male	1.33	0.87-2.04	0.20
HT	0.73	0.44-1.21	0.22
DM	1.58	0.99-2.50	0.052
Medication			
ACEI	1.20	0.62-2.31	0.58
ARB	0.55	0.31-0.96	0.04
SGLT2i	0.18	0.02-1.35	0.10
Statin	0.44	0.26-0.73	0.001

Abbreviations: ACEI, Angiotensin-Converting Enzyme Inhibitors; ARBs, Angiotensin Receptor Blockers; DM, Diabetes Mellitus; HT, Hypertension; PTH, Parathyroid hormone; SGLT2i, Sodium-Glucose Co-transporter-2 Inhibitor

Table S3. Determinants Associated with Ischemic Heart Disease (IHD) in CKD Stages 3-5ND According to our Cut-offs (PTH level ≥166 ng/L)

**Raw effect**	**HR**	**95% CI**	**p-value**
PTH ≥166 ng/L	1.59	1.05-2.40	0.03
**Model 1**	**HR**	**95% CI**	**p-value**
PTH ≥166 ng/L	1.66	1.05-2.63	0.03
Hypercalcemia	1.20	0.67-2.12	0.54
Hyperphosphatemia	0.96	0.56-1.63	0.87
**Model 2**	**HR**	**95% CI**	**p-value**
PTH ≥166 ng/L	1.80	1.11-2.90	0.02
Hypercalcemia	1.24	0.69-2.20	0.47
Hyperphosphatemia	1.07	0.61-1.88	0.81
Age ≥65 years	1.51	0.90-2.53	0.12
Male	1.33	0.86-2.03	0.20
**Model 3**	**HR**	**95% CI**	**p-value**
PTH ≥166 ng/L	1.82	1.12-2.94	0.02
Hypercalcemia	1.22	0.69-2.17	0.50
Hyperphosphatemia	1.02	0.58-1.79	0.94
Age ≥65 years	1.60	0.95-2.68	0.08
Male	1.35	0.88-2.07	0.17
HT	0.62	0.38-1.02	0.06
DM	1.36	0.86-2.15	0.19
**Model 4**	**HR**	**95% CI**	**p-value**
PTH ≥166 ng/L	1.65	1.02-2.66	0.04
Hypercalcemia	1.13	0.63-2.01	0.69
Hyperphosphatemia	0.99	0.57-1.73	0.97
Age ≥65 years	1.66	0.99-2.79	0.053
Male	1.30	0.85-2.00	0.23
HT	0.70	0.42-1.15	0.16
DM	1.56	0.98-2.48	0.06
Medication			
ACEI	1.32	0.68-2.55	0.41
ARB	0.57	0.33-1.01	0.053
SGLT2i	0.20	0.03-1.43	0.11
Statin	0.44	0.27-0.74	0.002

Abbreviations: ACEI, Angiotensin-Converting Enzyme Inhibitors; ARBs, Angiotensin Receptor Blockers; DM, Diabetes Mellitus; HT, Hypertension; PTH, Parathyroid hormone; SGLT2i, Sodium-Glucose Co-transporter-2 Inhibitor
